# Learning in a real-life escape room: an explorative study on the supervisory relationship in GP residency during the COVID-19 pandemic

**DOI:** 10.1186/s12875-023-02031-7

**Published:** 2023-04-01

**Authors:** Iris Meljes, Irene Slootweg, Vera Nierkens, Maartje van den Bogaard, Anneke Kramer

**Affiliations:** 1 Department of Public Health and Primary Care, Leiden University Medical Center, Hippocratespad 21, Zone V0-P, 2300 RC, PO Box 9600 Leiden, The Netherlands; 2 Department of Plant Pathology and Microbiology, Iowa State University, 1344 Advanced Teaching & Research Bldg, 2213 Pammel Drive, 50011 Ames, IA, USA

**Keywords:** Supervisory relationship, General practice, Residents, Supervisors, Disruptive, COVID-19, Workplace learning

## Abstract

**Background:**

The relationship between supervisors and residents plays a prominent role in the professional development of general practice (GP) residents. When disruptions occur in the normal course of healthcare, due to effects of e.g. war or emerging epidemics, we need to consider how this may affect the training of the next generation of general practitioners. As both supervisors and residents face new and unprecedented challenges that impact overall quality of the training. In this study, we examined the characteristics of the supervisory relationship in GP training during the disruptions early on during COVID-19. Our aim was to understand better how resident learning is affected in these circumstances, which is a first step in enabling supervisors, residents and faculty to anticipate disruptive situations better in the future.

**Methods:**

We conducted a qualitative case study with a constructivist approach. Seven GP residents at the start of their second placement, and their 10 supervisors participated in this study. Participants came from a University Medical Centre in the Netherlands. Semi-structured interviews were held between September 2020 and February 2021. The subjects were (1) interviewed individually about what they had learned regarding COVID-19, and (2) they were interviewed in supervisory pairs about how they had learned. Data were iteratively analysed; thematic analysis for (1) and template analysis in (2).

**Results:**

We identified notable changes in the supervisor-resident relationship attributable to COVID-19. Supervisors and residents were confronted with an all-encompassing uncertainty in the workplace, and disruptive changes in patient care and learning opportunities for residents. Supervisors and residents addressed these emerging workplace challenges through three types of collaboration, (1) getting the job done; (2) residents’ learning; and (3) collective learning. Each type had a different focus and distinctive characteristics of the supervisory relationship.

**Conclusion:**

With the outbreak of COVID-19, supervisors and residents were faced with disruptive uncertainty. In these circumstances, learning occurred not only between residents and their supervisors, but also with non-supervising GPs and assistants in collective learning. We propose to complement collective learning in the workplace with reflection between residents and supervisors at the training institution.

**Supplementary Information:**

The online version contains supplementary material available at 10.1186/s12875-023-02031-7.

## Background

The effects of war, climate change and emerging epidemics can create disruptive situations in healthcare [1] and medical education [2]. When these disruptions occur, it is necessary to consider what this means for the training of the new generation of doctors and determine whether residents training is still satisfactory [3]. In this study, we explored the nature of the supervisory relationship during COVID-19. General practice residents make the transition from novice doctors to fully qualified general practitioners (GPs) through participation in clinical care under the supervision of senior GPs [4–6]. Throughout resident training, the relationship with the supervisor is fundamental to residents’ acquisition of knowledge and skills, and the development of their professional identity [5, 7, 8]. This relationship enables them to manage clinical uncertainty and risk, maintain patient confidence, and take initial clinical responsibility for patient care, which is fundamental to functioning as autonomous GPs [6]. The supervisory relationship also plays an important role in balancing the tensions between patient safety and resident learning and autonomy in workplace learning [9, 10]. Workplace learning can be understood, according to Billet’s co-participation theory, as the interrelationship between workplace affordances and residents’ responses to these learning opportunities [11, 12]. Supervisors largely determine what residents are afforded to take on in clinical care [13, 14], and residents choose how they engage in these learning opportunities [15, 16]. Supervisors’ provision of opportunities and residents’ engagement with opportunities [17] are influenced by trust [18, 19] and supervisors’ and residents’ alignment on supervisory goals [20]. Recently, however, there has been a growing recognition of the benefits of encouraging the two-way nature of the supervisor-resident relationship. Not only to value the knowledge and skills that residents bring, yet also to acknowledge the lifelong learning journey of professionals [21, 22].

Nevertheless, our current understanding of how the supervisory relationship supports residents’ development and ensures patient safety is still underpinned by the premise that supervisors are medical experts and experienced doctors who combine clinical and educational activities [23–25]. However, in disruptive situations, such as disasters and unknown infectious disease epidemics [26], supervisors and residents face new and overwhelming professional challenges. Yet supervisors still need to guide residents’ professional development. The COVID-19 pandemic was such a disruption. During COVID, supervisors were no longer the medical experts in pathology [27] and had to reinvent their practices and patient care overnight [28]. Learning opportunities for residents were suddenly limited [29] and supervision went remote [30] due the restraints on physical contact [3]. This had implications for the relationship between supervisors and residents [31]. To understand the nature of the supervisory relationship during COVID-19, it is pertinent to consider the interactions between supervisors and residents in the workplace, the challenges they experienced and how this affected their learning processes while collaborating in this disrupted situation full of unknowns. To fully appreciate the changing dynamics of workplace learning, we used the work of Wiese et al. (2018) who created a comprehensive framework to understand learning processes and underlying learning mechanisms of workplace learning of residents and their supervisors [25].

## Theoretical background

The framework of Wiese et al. (2018) is embedded in sociocultural orientations to learning. This family of theories recognises that learning is inherently social. We learn from and through interaction with others and our environment. Learning is considered a transformative process. Learners transform their understandings, roles and responsibilities as they participate in the activities of a community [32, 33]. Specific theories that underpin the work of Wiese et al.’s are cognitive apprenticeship, Billet’s theory of co-participation, Communities of Practice and experience-based learning [25]. All theories consider learners’ increasing access to practice, where progress is achieved by reducing risk or increasing supervision [25]. Cognitive apprenticeship supports learning by enabling learners to acquire, develop, and use cognitive tools in authentic activity [32]. Central to Communities of Practice is that individuals adopt and acquire the roles, skills, norms and values of the culture and community through their participation, active engagement and increasing responsibility [33]. In experience-based learning learners engage in meaningful collaborative activities that contribute to patient care and personal and professional development as a doctor [33, 34].

Although Wiese et al. (2018) consider workplace learning between supervisors and residents in hospital settings, the three learning processes and underlying mechanisms they identified [25] correspond to the interplay between supervisors and residents in general practice training. The first process, *supervised participation* in the workplace, involves the mechanisms of supervisors’ *entrustment* of residents and residents’ *support-seeking* behaviour. Studies of supervisor- resident encounters in general practice show that supervisors need to be confident residents are able to provide safe patient care [35–38]. Whether and how supervisors’ confidence in residents is affected by a disruptive situation in clinical care is currently unknown. In the absence of disruptive situations, we know that this trust is built by gaining a sense of residents’ competence. This is influenced by residents’ help-seeking behaviour [25]. To ensure patient safety, residents seek help from supervisors when they feel uncertain or uncomfortable performing patient care independently [39, 40]. An important factor in residents’ help seeking behaviours is supervisors’ credibility [25, 37]. What is still unknown is how residents seek help in disruptive situations, as the credibility of supervisors may be affected as they are no longer more experienced than residents. In short, it is unknown how the mechanisms of entrustment and support -seeking are affected by a disruptive situation and, therefore, what supervised participation in the workplace looks like under these conditions.

The second learning process in the normal course of workplace learning is *mutual observation.* This is based on the mechanisms *of monitoring of the resident* by the supervisor and *modelling behaviour* by the resident. For the latter, residents observe the senior GP to integrate the senior’s behaviour in their own practice. When residents observe supervisors during a patient encounter, it supports them to make their own clinical judgements and manage uncertainty [23, 41, 42]. Supervisors monitor residents during the initial weeks of their placement through direct and indirect observation to maintain clinical oversight and establish confidence in residents [6]. At this point it is unknown how a disruptive situation which creates new challenges for supervisors, influences monitoring and modelling.

The third learning process between supervisors and residents in the workplace is *dialogue about practice* with the underlying mechanisms of *meaning-making* and *feedback*. Meaning- making aims to stimulate critical thinking and uncover underlying presumptions through shared clinical reasoning. Supervisors and residents do this by iteratively asking and answering questions [25]. In this context, feedback is seen as a mutual construction of residents’ performance and the means to improve it. This includes informal comments about their work, intertwined with discussions about patient care [25]. Supervisors’ ability to adapt their supportive style and feedback to residents’ learning needs [39, 43], their receptiveness to residents’ knowledge and their ability to stimulate residents learning through follow-up questions [38] affect the quality of the supervisory relationship and the learning opportunities for both resident and supervisor. At present, it is not known how these mechanisms of meaning- making and feedback are affected by new challenges presented to supervisors and residents in a disruptive situation.

Therefore, this study aims to contribute to the understanding of the supervisory relationship in a disruptive learning situation. An insight into the supervisory relationship will help to understand how residents’ learning is affected in a disruptive situation. This will also provide guidance on how to strengthen the supervisory relationship now and in future disruptive situations, and will contribute to workplace learning. The COVID-19 pandemic provided us with a good opportunity to investigate the supervisory relationship in a disruptive situation.

Our guiding research question is:

What characterizes the supervisory relationship in general practice residency during the COVID-19 pandemic?

## Methods

### Design

We took a qualitative approach to our research question, guided by principles of a constructivist paradigm, which means that we view reality as context-specific, socially constructed and experience-based, and therefore subjective [44, 45]. We chose a case study design because case studies generally delve deeply into relationships and processes, with the aim of unravelling the complexity of a given situation [46]. We took inspiration from Stake’s instrumental case study which he described as having “ a research question, a puzzlement, a need for general understanding, and feel that we may get insight into the question by studying a particular case” [47, 48]. Our case consisted of the supervisory relationship between pairs of GP supervisors and residents during COVID-19, which is a naturally occurring and bounded situation, typical of a case study [44, 46].

Consistent with the chosen research approach and design, we sought to understand residents’ and supervisors’ perceptions of the supervisory relationship in regard to workplace learning during the COVID-19 pandemic. We first explored their workplace learning experiences during the outbreak of the pandemic. To recognise both the subjective experiences and insights of residents and supervisors and the importance of the interaction between supervisor and resident in constructing meaning [44, 45, 49], we chose to conduct two rounds of semi-structured interviews. Due to restrictions on human contact at the time this study was conducted, we were unable to use observation as a method of data collection. In the first round of interviews, we asked residents and supervisors individually about their meaningful learning experiences related to the COVID-19 outbreak. In the second round, we asked resident and supervisor pairs about the activities undertaken and their roles and contributions to these learning experiences. By conducting joint interviews and having resident and supervisor pairs respond to each other’s experiences and insights, we expected to gain an in-depth understanding of supervisory relationships during COVID-19 [44]. We received ethical approval to conduct this study from the Educational Research Review Board of the Leiden University Medical Centre. The first interview round was held between September and November 2020 and the second between January and February 2021.

### Setting and participants

This exploratory study is part of a larger research project aimed at designing an evidence-informed, innovative postgraduate programme in general practice. In general, this project encouraged the development of medical leadership skills for residents. It included several cohort studies in a design-based research approach. Residents registered themselves for the innovative GP programme. They were free to choose not to participate in the different sub-studies. All residents in the third cohort of the innovation project participated in the present study.

Participants were seven general practice residents and their 10 supervisors. The residents were aged between 31 and 35 years, four of them were women and three were men. The supervisors were aged between 37 and 62 years. Six of them were women and four were men. The supervisors had between three to seven years of experience in training residents. Participants came from one University Medical Centre in the Netherlands. The residents had completed the first phase of general practitioner training, which consists of 12 months of general practice placement and 6 months of emergency training. As their final phase of training, the 18-month general practice placement, coincided with the start of the COVID-19 pandemic, these residents and their supervisors could provide us with an insight into the supervisory relationship during the outbreak of COVID-19.

### Research team

The research team consisted of an educationalist with no prior background in medical education and PhD student (IM), an educationalist and specialist in medical education (IAS) an educationalist specialized in STEM (MvdB), a health scientist (VN) and a professor in general practice focused on medical education and initiator of the innovative GP program (AK). None of the researchers were directly involved in the training of residents or supervisors. At every stage of the study - design, execution, analysis, writing - everyone’s expertise was brought to bear on the findings.

### Data collection

**Fig. 1:**
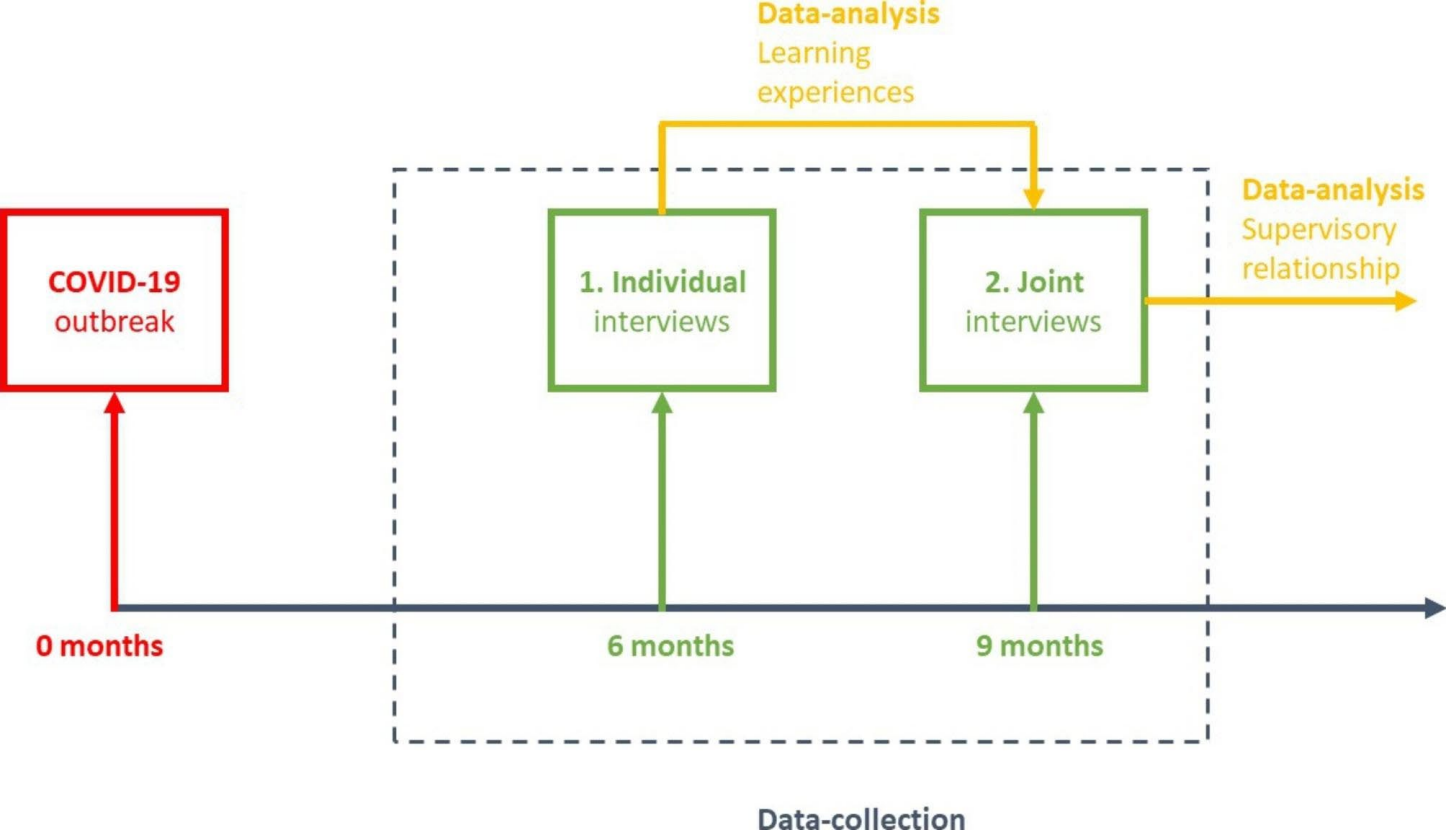
Overview of data collection and data-analysis process

An overview of the data collection is provided in Fig. 1. Prior to each interview round the main researcher held a trial session with volunteer(s). These trial sessions were observed by the second author and discussed afterwards to improve the main researcher’s interview skills and the interview guide.

In the first round we gathered information about *what* residents and supervisors had learned concerning the COVID-19 outbreak (see Appendix 1 for the individual interview guide). We asked participants for their three most important learning experiences. We defined learning experiences as: the knowledge and skills that someone has gained, and the change in someone’s attitude, from doing something for a period of time and the process of gaining them [50].

For this first round, we chose to interview one on one to create a safe environment in which participants would feel free to elaborate on their experiences.

In the second round, we used the results of the first round to explore *how* participants had learned. We were specifically interested in whether and how the supervisory relationship had played a role (see Appendix 2 for the joint interview guide). Hence, we chose to interview resident-supervisor pairs and encouraged them to react to each other’s reflections [51]. All interviews were held online, were recorded, and transcribed verbatim.

### Data analysis

#### First round

Since learning experiences concerning the COVID outbreak were novel and not previously described we used an inductive approach to data analysis. Using thematic analysis [52, 53],we constructed basic themes from the data, which were grouped into organising themes, and finally aggregated into global themes [54]. We used an Atlas.ti software package to support data management and coding. The analysis was conducted in the following way. IM, MG, and VN familiarised themselves with the data before starting independent coding. Basic themes were derived from the data using In Vivo codes to accurately capture participants views. IM coded all 17 transcripts and MG and VN independently coded 13 transcripts in total. Dilemmas concerning coding were discussed with MG and VN until agreement was reached. After coding basic themes, IM, VN and IAS clustered codes representing similar issues into groups of organizing themes. Through additional rounds of discussion IM, IAS, VN, and AK constructed global themes capturing common learning experiences pertaining to COVID-19. We selected global themes that reflected learning experiences relating to the interplay between supervisors and residents for the second interview protocol.

#### Second round

The transcripts of the 10 joint interviews were analysed through template analysis [55]. Template analysis can be seen as a form of thematic analysis. It is a method for identifying, analysing and reporting patterns or themes within data. In template analysis, it is possible to start with some a priori themes identified in advance as likely to be useful and relevant to the analysis [55]. To better our understanding of the supervisory relationship during COVID-19, we found it particularly important to look for the three learning processes between supervisors and residents – supervised participation, mutual observation, dialogue about practice - and their six underlying mechanisms – entrustment & support seeking, monitoring & modelling, meaning-making & feedback - described by Wiese et al. [25]. We used these as a priori themes. This allowed us to combine the use of deductive, theory based, and inductive, data driven, coding [52].

Initially,the apriori codes were used as a template for data analysis. However, we did not restrict ourselves to these codes and kept an open mind to identify new patterns and codes to describe best the interplay between resident-supervisor pairs under disruptive circumstances. IM coded all 10 joint interviews. Seven interviews also coded by either VN or MH. After 2 transcripts were independently double -coded, codes were compared and dilemmas were discussed until consensus was reached. The initial template was adjusted iteratively, and coded transcripts were adapted. The final themes were created through additional rounds of discussion between IM, IAS, VN, and AK.

## Results

Before we explain what the supervisory relationship during the disruptive period of COVID-19 looked like, we first consider the learning experiences deemed significant for this period. The insights gained from these learning experiences into the nature of the clinical workplace during the pandemic are essential for understanding the interplay between residents and supervisors during this period. From the data,  we constructed four global themes of learning experiences related to the supervisory relationship : (1) disruptive uncertainty; (2) patient care; (3) learning opportunities of the residents; (4) supervision. In relation to the fourth theme, supervisors reported they learned much about providing supervision and residents about receiving supervision. We included these results in the second part of the results section, where the emphasis is on the supervisory relationship.

### Learning experiences

#### Disruptive uncertainty

The outbreak of COVID-19 immediately and abruptly confronted supervising general practitioners and residents with a high extent of uncertainty in the workplace:

“This was something no one had ever experienced, not even my 7 colleagues who are in their 60s” (Supervisor 1).

Supervisors and residents perceived high levels of *unpredictability* in their work. This manifested in two ways. First, the evident lack of medical knowledge about the clinical symptoms, the course of the disease and effective treatment.

“You learn about the medical content from your patients because it is a new disease, so you don’t know what the recovery process will be like. In the beginning, you are not very clear about how it presents itself; we didn’t know the first symptom of infection could be the loss of smell and taste” (Supervisor 7).

Second, the contamination rate of COVID-19 was unknown and unpredictable. Consequently, some of the supervisors and residents became anxious for their own or their family’s health and either wore extensive protective equipment or worked from home. In addition, the risk of contamination affected the way patient care was organized, because common routines to see patients became potentially risky. However, national policies and local guidelines were constantly updated, and protective equipment was insufficient, especially at the start of the outbreak. As a result, each GP practice had to evaluate the situation and determine its policies and procedures. One protective measure taken in all GP practices was the restriction of physical examinations. One resident voiced the confusion and uncertainty they experienced as follows:

“To examine patients, when do you use face masks, gloves? And when do you not use them? Who do you invite to the consultation hours? Who is not invited? These are really tricky things. I think that goes for everyone” (Resident 1).

.The profound uncertainty disrupted workplace routines. This created novel learning opportunities as supervisors and residents faced new challenges.

“In the beginning you still had to come up with creative solutions” (Resident 5).

#### Patient care

Much of the delivery of patient care had to be reinvented as physical consultations were largely replaced by telephone triages and consultations. Telephone triage became an essential part of the daily work of residents and supervisors. They had to assess whether the physical examination of patients could be carried out safely. If a patient was infected with COVID, they decided whether the patient should be referred to the hospital or whether they could check in with the patient regularly via telephone consultations. Residents commented that they initially found it “difficult” and “burdensome” to decide how ill a person was over the phone, with little medical knowledge about the symptoms of COVID-19. This discomfort was expressed by a resident:

“suddenly every tool you had was knocked out of your hands” (Resident 2).

However, residents and supervisors learned to make these assessments over time:

“I learned a lot from assessing patients’ complaints and symptoms over the telephone and acting on the findings, and it pays off when you do telephone consultations” (Resident 5).

Over time, as residents and supervisors gained insight into the symptoms of COVID-19, they learned to accept the limited role they had in the care for COVID patients:

“If they [patients] are not sick enough then there is not much you can do for them, and it is really a matter of waiting to see how things develop, and that is a process of learning to let go” (Resident 7).

As supervisors and residents became accustomed to providing patient care over the phone, they began to recognize some advantages for patient care. Some supervisors who were initially sceptical about telephone consultations learned that

“while we were thinking ‘we can’t do anything anymore because everything has to be done by telephone’, we noticed that 95% of things do work out using the telephone. That the world does not end when you are severely limited when it comes to seeing patients physically” (Supervisor 9).

In addition, residents and supervisors described the advantages of digital tools for patient care:

“We mainly use e-consulting, we make use of pictures [of symptoms sent by patients], and we do much more by phone. Covid brought these developments to the practice, and we still notice the benefits. We can easily take care of little things by using photos or by providing telephone advice” (Supervisor 8).

#### Learning opportunities for residents

The uncertainty and the challenges in patient care blocked routine learning opportunities, such as chronic elderly care. Yet,  it also allowed for new learning opportunities. Unable to revert to how they usually started the training of a new resident, supervisors had to find new ways of training residents. Many focussed on trying to create sufficiently diverse learning opportunities:

“My resident worked in the COVID facility [in a hospital] several times from the beginning. I felt she should learn the things that can be specifically learned right now, so I tried to seize really the moment” (Supervisor 1).

Most of the residents learned to accept that the final part of their training period was different than they had envisioned it to be. They learned to utilize effectively the new opportunities:

“I’m sure there are things I have not been able to learn as a result. However, it really has been a unique opportunity to experience this pandemic as a resident instead of a qualified GP or locum” (Resident 7).

In sum, we described three themes of learning experiences that were significant during the pandemic as they specify the unique challenges faced by supervisors and residents. We used these themes to determine characteristics of the supervisory relationship during the COVID-19 outbreak.

### The supervisory relationship

Collaboration between supervisors and residents proved to be a key element in their relationship during the outbreak of the pandemic. We identified three types of collaboration, each focussed on different workplace challenges and achieving different goals. We label these three types of collaboration as: *getting the job done, residents’ learning, and collective learning*. For each type of collaboration, we describe the characteristics of the supervisory relationship.

#### Collaboration focussed on getting the job done

The aim of this type of collaboration between supervisors and residents was to continue patient care under disruptive circumstances, or ‘ getting the job done’. To this end, supervisors transferred part of patient care to their residents as if they already were qualified colleagues. Residents characterized their learning process as ‘learning by doing’. Supervisors seemed to entrust them more readily with the care than they would have under normal circumstances.

“When a resident joins our practice, at first you need to find out who this person is and how things go. Yet, because I was working elsewhere [in the emergency organization for local Covid care], the resident was in charge right away and I think that actually got the training off to a flying start” (Supervisor joint interview (ji) 9).

Supervisors did acknowledge that residents were not yet proficient colleagues as they continued to monitor residents’ ways of thinking, their actions, and decisions. For instance, supervisors continued to monitor their residents by keeping track of their calendars.

“You see in your calendar what is happening [...] you see the patient’s name in italics and when I click on it, I see what happened. And 9 times out of 10 it is just a second, and for some patients you ask the resident why they handled it in a certain way and how they think we should continue care. So, the difficult cases do come up along the way” (Supervisor ji 3).

In addition to monitoring, the supervisors also provided a safety net for their residents. As experienced colleagues, they continued to be available for support or to discuss immediate patient care issues.

“The fact that you can always call to consult [with the supervisor about telephone consultations], and the possibility to discuss afterwards what went well or what didn’t, gives you peace of mind” (Resident ji 3).

Residents’ support-seeking behaviour when faced with challenges they did not feel comfortable dealing with themselves was also an important element in this type of collaboration. The combination of supervisors’ entrustment, monitoring and provision of a safety net, and residents’ support-seeking behaviour allowed residents to take on the role of new yet qualified colleague right from the beginning.

#### Collaboration focussed on residents’ learning

In GP practices where physical consultations were restricted for months, learning opportunities for residents were not self-evident. In these circumstances residents and supervisors were aware of the challenge and the need to find sufficient learning opportunities for residents. We found three ways in which supervisors and residents collaborated to promote residents’ development. In general, supervisors initiated learning opportunities, yet not always, and residents chose how to utilize these opportunities. First, supervisors and residents tried to *use existing possibilities optimally*. For example, supervisors invited their residents to participate structurally in discussions about management.

“I tried to involve my resident in many issues, such as how we make decisions about the practice” (Supervisor ji 8).

Residents used the available opportunities to their advantage. They used their position as learners to actively participate in team deliberations without the burden of accountability:

“Now something really has to change, and then you see what happens when […] a walk-in consultation hour suddenly stops and 11,000 patients have to be informed. […] It is great to be able to observe this, especially since you are not responsible for it, you can have opinions and think about it without it having many consequences” (Resident ji 3).

Second, supervisors and residents *created additional learning opportunities*. In some cases, supervisors transferred their consultation hours to their residents in order to increase their exposure to patients :

“You gave me some of your consultation hours. Then you did the telephone consultations with the assistants so that I could see more patients” [resident addresses supervisor during the interview] (Resident ji 8).

In other cases, residents initiated a conversation with their supervisor about the need for more patient consultations.

“I did say that I needed to see more patients. I want to see more patients so I can develop professionally. I was worried that I might miss opportunities to learn. And my supervisors agreed that they had to arrange more patient contact for me” (Resident ji 5).

Third, supervisors created *new learning opportunities specifically related to COVID-19*. Residents made use of these opportunities.

“(My supervisor) involved me in what was going on with the whole organisation (of COVID care in the area). I was able to work at the COVID facility soon after it became a thing, which was exciting, and also very cool in the beginning. We were in those COVID suits with large masks straight away. […] those are advantages and I think we really used them” (Resident ji 9).

In addition, amidst the hectic workplace, supervisors and residents had to make time for reflection and discussion about residents’ learning. Whereas supervisors and residents used to have dedicated reflection sessions to discuss issues ranging from patient care to residents’ professional development, these ‘reflective learning conversations’ were no longer self-evident. Residents and supervisors placed different levels of importance on these learning conversations. The amount of time allocated to these conversations varied from practice to practice. Some supervisor-resident pairs used these conversations as an anchor point to structure the residents’ training in an otherwise chaotic time. In other practices, the ‘reflective learning conversations’ were abandoned due to the hectic start of the training and they were not implemented later.

Finally, this type of collaboration did not include mutual observation. Nor did we find signs of modelling.

#### Collaboration focussed on collective learning

This type of collaboration occurred between all team members of the GP practice, not just between supervisors and residents. The aim was to gain a better understanding of the disease and its impact on patient care and the organisation of patient care. Residents, supervisors, non-supervising GPs and other team members shared information and developed strategies to adjust effectively the organisation of patient care. All team members had equal positions in this collective enterprise. The following quote from a supervisor illustrates the collective team spirit at that time:

“We were absolutely no better informed than, well, no more than the doctor’s assistants. I mean everyone started from zero and that also made it enjoyable. It is as if you have to solve a problem together in an escape room. Yes, everyone contributes something […] it is a kind of new problem that everyone is totally focussed on” (Supervisor ji 8).

As supervisors, residents, and non-supervising GPs experienced similar challenges in patient care, including telephone triage and consultation, they shared experiences and exchanged information to improve their diagnostic skills over the telephone. The following quote from a resident illustrates that residents discussed their doubts concerning clinical activities with several GP colleagues, rather than restricting themselves to their supervisor:

“Because everyone was learning (triage over the phone), […] so we talked a lot in the coffee room […] you just discussed a lot with each other like: this was difficult for me. We are a huge team […] and we discussed so much in the early corona meetings, things like (following up on) ‘how did that go?’ and sharing tips and tricks with each other (Resident ji 1).

As residents engaged in collective meaning-making activities, they quickly became a part of the team.

“I think you […] become part of a team much faster if a new situation is created for everyone, because then everyone has to find a new position, like OK, how are we going to do this. So you definitely feel more at home and you get to know everyone quicker” (Resident ji 8).

Our data do not indicate whether the information shared, and the knowledge created collaboratively with the team circled back to the supervisory relationship, for example, in reflective learning conversations. Nor did we find clues about feedback on residents’ performance.

In conclusion, collaboration between residents and supervisors early in the pandemic was manifested in three types, each containing distinctive characteristics of the supervisory relationship. In the first type *getting the job done*, residents took on the role of starting yet qualified colleague, while supervisors acted as senior colleagues providing a safety net. In the second type *residents’ learning*, the resident’s role was that of the learner who needed enough learning opportunities to develop competencies, while the supervisor acted as a facilitator of learning. The third type of collaboration, *collective learning*, went beyond the supervisor-resident dyad. Residents and supervisors were both team members who learned about the pandemic in the same way as the other team members. All supervisor-resident pairs used all three types of collaboration depending on the challenges they faced.

## Discussion

### Principal findings

In this study we explored the impact of a disruptive situation, i.e. the COVID-19 pandemic, on the relationship between GP residents and their supervisors. We found that the disruptive uncertainty, the extensive consequences for the patient care and the impact of learning opportunities for residents resulted in three types of collaboration. Within the supervisory relationship, supervisors and residents collaborated to get the job done and to facilitate residents’ learning. Beyond the supervisory relationship, collective learning emerged as supervisors, residents and other team members collaborated in information sharing and joint meaning-making to enhance their understanding of the situation and to improve patient care. While the first two types of collaboration, getting the job done and residents’ learning, are typical for workplace learning, the extent to which they occurred was different. Getting the job done became more prominent as residents were encouraged to quickly work independently. In contrast, creating learning opportunities for residents required more attention and inventiveness since the regular training structure fell away. The third type of collaboration, collective learning, i.e. learning with and from each other, including non-supervising GPs and the entire team, has received little attention in the literature on general practice training. Our study contributes to the understanding of the supervisory relationship in GP training, by providing insight into the characteristics of this relationship during a period of major and unprecedented change.

As we discuss our findings in the light of other literature, we will begin with the impact of uncertainty on the supervisory relationship during the COVID-19 pandemic, before we continue with the types of collaboration and the underlying interaction patterns that we found.

### Uncertainty

Clinical uncertainty is a key aspect of general practice as the entry point to the healthcare system [6, 56]. Residents learn to manage and tolerate this uncertainty by observing how their supervisors deal with uncertainty. Residents develop the same strategies for responding to uncertainty as their supervisors [41, 42]. However, the type of uncertainty we addressed in this study is pervasive and disruptive - causing problems so that something cannot proceed normally [57]. The scope of the uncertainty we found was not limited to clinical uncertainty, yet was much broader. Supervisors, residents and other team members were faced with the unpredictability of the disease and its impact on care delivery. As a result, even supervisors had to learn to manage this type of uncertainty. Uncertainty was therefore a general and shared lack of clarity about clinical activities and the organisation of patient care, rather than a ‘state of mind’ of an individual, often the resident [58]. Interestingly, residents and supervisors used strategies to cope with uncertainty similar to those described by Han and colleagues (2021), namely [58], i.e. (1) ignorance-focussed; (2) uncertainty-focussed; (3) response-focussed; and (4) relationship-focussed. They reduced their ignorance by, for example, seeking medical information. They changed their response to uncertainty by learning to accept their limited influence on patients with COVID disease. Finally, they shared doubts or discomfort they felt with their team, a ‘relationship’-focussed strategy. Our study adds collective strategies, such as shared meaning-making, to the individual strategies used by supervisors and residents to deal with disruptive uncertainty.

### Collaboration and interaction patterns

The influence of disruptive uncertainty is reflected in the types of collaboration and underlying patterns of interaction between supervisor and resident pairs. The first type of collaboration, ‘getting the job done’, involved the underlying interaction patterns as described by Wiese et al. (2018) of supervisors’ monitoring and entrustment, and residents’ help seeking behaviour. We found that supervisors immediately entrusted residents with patient care. While entrustment in general practice residency develops rather rapidly, holistically and presumptively [6], the pace by which entrustment was awarded here was remarkable. The residents, in turn, accepted the invitation to patient care as though they were already qualified colleagues. This type of collaboration facilitates residents’ participation and active engagement in the professional community as they took on increasing responsibility for patient care [33, 59]. Sfard’s metaphor of learning as ‘participation’[60] is appropriate here. It conceives of learning as a process of meaning construction and identity formation through participation in community activities [32]. The second type of collaboration, residents’ learning, involved supervisors creating sufficient learning opportunities for residents to develop the competencies required for the diverse range of patients in primary care. This resembles Sfard’s metaphor of learning as ‘acquisition’ [60]. In this metaphor learning is seen as acquisition of knowledge, skills, and attributes that are ‘owned’ by the individual [32]. We did not find any signs of supervisors demonstrating their expertise nor of residents integrating their supervisors’ modelled skills. This might be because the supervisors did not have more specific expertise than the residents, or because of the limitations in interpersonal contact, which prevented supervisors and residents from seeing patients together.

The third type of collaboration, collective learning, involved residents, supervisors and other staff creating an understanding of the impact of the pandemic and developing new knowledge for patient care and policy. They did this through a collaborative process of meaning making and problem solving. This type of collaboration facilitated the joint creation of knowledge. This resembles Paavola and Hakkarainen’s metaphor of learning as ‘knowledge creation’ [61]. In this metaphor, learning is seen as the collaborative development of activity in response to challenges.

In short, our findings show how supervisors and residents collaborate in workplace learning during a disruptive situation. Although we found some similarities with the learning processes and interaction patterns described by Wiese et al. [25], other features of the supervisory relationship were highlighted during the pandemic. This adds to our understanding of the relationship between supervisor and resident in workplace learning.

### Collective learning

The most notable finding for us is that of collective learning, as it is a clear complement to workplace learning between supervisors and residents and the supervisory relationship in times of disruption. However, it is not a surprising finding. Collective learning appears to be an intuitive response to new or complex challenges [62], and during the first outbreak of COVID-19, no one had more expertise than anyone else. Also, the shared challenges supervisors, residents and other staff faced during the pandemic created a sense of belonging, eliminating the sense of hierarchy that could hinder collective learning [63]. Collective learning is internally generated by a team who are willing to learn and who are aware of the importance of finding solutions to practice problems [63]. Because it is often implicit, collective learning is frequently unnoticed by professionals [63]. The disruptive situation of COVID-19 made collective learning explicit.

### Strengths and limitations

This study has several methodological strengths and some limitations. First, to our knowledge, this is one of the few studies to explore the supervisory relationship under conditions where the supervisor is not the expert. Second, our research design allowed us to build on participants’ actual learning experiences and as such we developed a rich understanding of the impact of COVID-19 on the workplace and of the supervisory relationship therein. The limitations of this study are that we had to narrow our methods to online interviews. Observations could not be included due to the restrictions on interpersonal contact at the time of our data collection. Our study might have benefited from observations as a means for triangulation of the data that could potentially lead to even richer data.

### Implications for practice and further research

This study provides insights into the supervisory relationship in relation to workplace learning in disruptive times and has clues to improve the training of future general practitioners. First, collective learning in teams occurs in response to major common challenges. It is ad hoc and implicit [62, 63]. To further enrich the learning experiences, collective learning can be complemented by reflection. Reflection creates a better understanding of oneself and the situation, which helps to inform future actions [64].

Reflection can be promoted in the training institution during a release day by encouraging residents, supervisors and, possibly, other staff to discuss and reflect together on situations and to formulate lessons learnt. This may be done retrospectively, after the turmoil in the GP practice has subsided. Secondly, it is important that residents have sufficient and varied learning opportunities to develop as GPs. Whether this is possible in a period of disruption depends in part on the learning opportunities available in the workplace. The training institution can play a mediating and facilitating role by bringing together different supervisors to share or create opportunities.

We also see several avenues for further research. We need a better understanding of less established forms of clinical workplace learning, including collective learning, bi-directional learning and reverse mentoring [21, 22] - which we anticipate will become more common in the future. Situations where the supervisor is no longer the expert are expected to become more common. We expect residents will be more familiar with eHealth applications and ways to interact with patients through social media than their supervisors, and for residents to take on a mentoring role. To further our understanding of learning between supervisors and residents on such topics, an observational study could be conducted to explore what kind of learning occurs, its nature, and its barriers and facilitators.

## Conclusion

The supervisory relationship was characterized by three types of collaboration during the outbreak of COVID-19: (1) getting the job done, (2) residents’ learning and (3) collective learning. Collective learning complements our general understanding of the supervisory relationship and workplace learning between supervisors and residents. Collective learning occurred when supervisors of residents and other staff were faced with the disruptive effects of COVID-19, where supervisors were no longer the experts. Situations where supervisors are no longer the experts are likely to become more common with the introduction of e-health applications and interaction with patients through social media. Therefore, a better understanding of collective learning and other less established forms of clinical learning in the workplace is needed.

## Electronic supplementary material

Below is the link to the electronic supplementary material.

Supplementary Material 1

Supplementary Material 2

## Data Availability

The datasets used and analysed during the current study are available from the corresponding author (Iris Meljes I.meljes@lumc.nl) on reasonable request.
