# Supplementary material for: Learning in a real-life escape room: an explorative study on the supervisory relationship in GP residency during the COVID-19 pandemic
Source: BMC Prim Care. 2023 Apr 1;24:87. doi: 10.1186/s12875-023-02031-7 (PMC10066972; doi:10.1186/s12875-023-02031-7)
Supplement: Supplementary file 1 — Supplementary Material 1 [file 12875_2023_2031_MOESM1_ESM.pdf]

Protocol individual interview

Interview procedure

In this study we are interested in how you, as GP resident/supervisor, learn new things as a (future) general practitioner and if and how your supervisor/GP resident plays a role in this. We have chosen Covid 19 as an example of learning something new.

In this interview, I am going to ask you some open questions about how and what you have learned about Covid 19 and discuss what were important learning moments for you. We will take one hour for this interview. I will record this interview to transcribe it for research purposes. This interview will be treated confidentially, and will not be traceable to you.

Questions

1. How do you look back on the past months of your residency ?
    - How has Covid 19 played a role in this?

I will now ask you some questions about the learning moments that have contributed to the feeling of confidence that you know what to do with a patient who may have corona.

2. Write down on a (digital) post-it the learning moments that have been important for you.

How many are there? If there are more than three, we will start with the three most important ones.

Now we are going to put those learning moments on a graph. The y-axis of the graph indicates how you experienced the moment in terms of positive or negative. The x-axis shows when the learning moment took place, the time is shown in months.

3. Place the 3 most important learning moments in the chart.

Draw a line through these learning moments to connect them and make the learning process, or learning over time, visible.

4. Explain what happened in these learning moments.

- What was the role of your GP resident/supervisor in this?
- Is that role different now than it was at the beginning? Describe the difference.
- How did you learn from this moment?

5. How do you experience the relationship with your GP resident/supervisor?

End

Thank you very much for participating in this study. I will soon invite you for a follow-up. You will then be interviewed together with your trainee to discuss further how the trainee-teacher relationship contributes to learning new things as a (future) GP.

Definition list:

A **learning moment** is a situation from which something can be learned. Also called a learning experience or a moment/situation of surprise and amazement. Learning moments are often recognised as such afterwards, when things have (almost) gone wrong.

Experience of learning moment: manner in which learning moment was experienced. The extent to which the learning experience was positive or negative is indicated on the y-axis.

Learning process: learning in time. Is mapped by connecting the learning moments in the graph to create a line.
