# Supplementary material for: Learning in a real-life escape room: an explorative study on the supervisory relationship in GP residency during the COVID-19 pandemic
Source: BMC Prim Care. 2023 Apr 1;24:87. doi: 10.1186/s12875-023-02031-7 (PMC10066972; doi:10.1186/s12875-023-02031-7)
Supplement: Supplementary file 2 — Supplementary Material 2 [file 12875_2023_2031_MOESM2_ESM.pdf]

Protocol joint interview GP resident- supervisor

Introduction

This joint interview is part of a study into the relationship between supervisor and GP resident when learning new aspects of the GP profession, particularly Covid-19. The aim of this interview is to gain insight into the *experiences of the supervisory relationship when learning new things.*

The previous 17 individual interviews were used as input for this interview. We coded the individual interviews anonymously and extracted four themes from the data.

The 4 themes we will discuss during this interview are:

1. Coping with an uncertain world
2. Consultations by telephone
3. Creating learning situations
4. Giving and receiving supervision

We are not looking for right or wrong answers, we are looking for your experiences in learning.

This interview will also be coded anonymously to extract themes that say something about the experiences of the supervisor relationship during learning about and during the Corona pandemic.

We will report back to you as soon as we can.

The interview lasts 60 minutes and because it is a joint interview it is important that you let each other finish and use the opportunity to discuss with each other.

I will record the interview from now on.

START

Just think about how you are sitting here, how you feel about being interviewed together.

- How do you feel about being interviewed together?

We go back to the outbreak of COVID-19 in march 2020. From the earlier interviews, the theme came up:

1. Uncertain world

A quote from an interview:

Everybody has that uncertainty, nobody knew how and what. So just here in the practice they made choices, they started organising things. In the beginning I thought, that' somewhat exaggerated.

Based on literature, we know that the characteristics of the supervisor and the resident are important in the supervisory relationship. That is why we will take a moment to look at your characteristics.

- How did you experience the other person in such during the uncertain situation?
- How do you see yourself in an uncertain situation?
- To what extent did you recognize these characteristics in the other?

2. **Consultations by telephone**

Remember, you could no longer rely on the diagnostic skills you normally use. You had to learn to recognise symptoms without being able to see patients and only rely on what you hear. It was also different for patients, who could no longer just go and see a doctor.

Among other things, it has been said about telephone consultations:

All your tools are knocked out of your hands (resident)

It was, of course, also a period in which I myself [as a supervisor] learned a great deal about telephone consultations, about video consultations, about what is useful and what can be done by telephone and what turns out to be very difficult to do by telephone in practice.

- How did you learn to do telephone consultations during the outbreak of COVID-19?
    - What did telephone consultations mean for learning shared decision making? How did you deal with it?

- *Have you done things together with regard to telephone consultations? What did you share with the other person?*
- What role did the supervisor/GP resident play in learning how to conduct telephone consultations?
    - What did the supervisor/ resident do?
    - What did you discuss together?
    - Have you made arrangements for telephone consultations?

Topics

- Learning objectives > how did learning objectives play a role in learning
    - How do you know what to learn?
    - Did you include competences and goals?

3. **Creation of learning situations during the outbreak of the pandemic.**

Based on the previous interviews it appeared that there were concerns about the diversity of patient problems that residents saw. It became apparent that attempts were made to make the training situation as diverse as possible

despite the circumstances. Learning opportunities were sometimes sought outside the own practice.

Quote:

Yes, as a supervisor, I had to understand him and to see exactly who he was as a doctor. You're a bit, well, you base your activities on your experience in recent years, we always first had residents do some consultations, then we can see who they are, but there were actually almost no consultations to do.

- How did you ensure that there was enough to learn during the outbreak of the pandemic?
- What are your experiences with learning together?
    - *Have you dealt with things together? What have you shared with the other?*
    - *Supervisors also learned new things, how did these affect the learning situation of the AIOs?*
- What role did the supervisor/GP resident play in creating the learning situation?
    - What did the supervisor do?
    - What did you discuss together? What did you need each other for?
    - Have you made arrangements regarding creating learning situations?

4. Giving and receiving supervision

The possibility for conscious training differed among the supervisor-resident pairs, as did the frequency and perceived quality of the learning conversations.

Quote: *there was no learning conversation for the resident, it was chaotic, well, the time and attention for the resident, yes, not what it should be I think*.

Let's go back to the features of resident and supervisor.

- What went well between you as resident and supervisor during the outbreak of COVID-19?
- And what took more effort/ more attention when it comes to learning new things during the outbreak?
- How do you characterize the supervisory relationship in uncertain times?

End

- Are there any relevant topics that have not been covered?
- How did you find the interview?
